# Cryptocurrency in sport: a thematic review

**DOI:** 10.3389/fpsyt.2025.1745490

**Published:** 2026-01-28

**Authors:** Xinliang Zhou, Yunfei Tao, Li Huang, Haodong Tian, Haowei Liu, Xing Zhang, Mingyue Yin, Zhenhuan Wang, Hansen Li

**Affiliations:** 1 School of Physical Education, Xihua University, Chengdu, China; 2 School of Sport Training, Chengdu Sport University, Chengdu, China; 3 Faculty of Psychology, Southwest University, Chongqing, China; 4 College of Physical Education, Southwest University, Chongqing, China; 5 Department of Physical Education and Sport, Faculty of Sport Sciences, University of Granada, Granada, Spain; 6 School of Athletic Performance, Shanghai University of Sport, Shanghai, China; 7 Institute for Health and Sport (iHeS), Victoria University, Melbourne, VIC, Australia; 8 School of Physical Education, Sichuan Agricultural University, Ya’an, China

**Keywords:** addiction, bitcoin, blockchain, fan tokens, gambling risk

## Abstract

**Introduction:**

Blockchain-enabled products (e.g., cryptocurrencies and fan tokens) have rapidly expanded across professional sport, but the research landscape remains dispersed across finance, marketing, information systems, and sport management.

**Methods:**

This study conducted a thematic review of Web of Science Core Collection records supplemented by snowball searching, yielding 30 English-language peer-reviewed studies published between 2019 and 2025.

**Results:**

Based on the included titles, we mapped how the literature has developed and what it collectively implies for sport organizations, platforms, and consumers. Five recurring strands were identified: (1) fan tokens and sport cryptoassets as financial assets, emphasizing volatility, spillovers, and sensitivity to sport- and crypto-market events; (2) adoption, identity, and engagement research explaining why supporters buy/hold tokens, participate in voting, and engage in advocacy; (3) computational and platform-data approaches (e.g., sentiment/discourse analyses and poll/voting participation patterns) to quantify online engagement and market narratives; (4) blockchain applications and governance, including stakeholder-oriented discussions and ethical critiques regarding value creation, transparency, and power asymmetries; and (5) gambling-like risks and addiction-related correlates, highlighting the convergence of trading, betting-like dynamics, and potentially harmful consumption.

**Discussion:**

Limitations include dependence on WoS-indexed English-language publications, topic and context concentration (especially European football and major platforms), and heterogeneity in study designs and outcomes that precludes comprehensive data synthesis. Future research should broaden contexts beyond dominant sports/regions and use stronger longitudinal or quasi-experimental designs to test mechanisms and harms.

## 1 Introduction

Blockchain is a decentralized distributed ledger technology that enhances data security and transparency through cryptography. By enabling information to be shared and verified across multiple nodes, it reduces reliance on a central authority and improves resistance to tampering. Blockchain has been applied in areas such as financial services, risk management, the Internet of Things (IoT), and public services (1). Among its applications, cryptocurrencies are the most recognized, using cryptographic algorithms to secure transactions and support pseudonymous transfers. Examples include Bitcoin, Ethereum, Dash, Litecoin, and Monero (2), which are used for online payments, investment, and value storage.

Unlike traditional currency transactions, cryptocurrencies operate without intermediaries such as banks, which may increase transparency and facilitate peer-to-peer transfers (3, 4). Since the introduction of Bitcoin in 2008 (5), cryptocurrency adoption has expanded rapidly, and the number of available crypto assets has grown substantially (6).

Beyond cryptocurrencies designed primarily for financial transactions, new blockchain-based digital assets have emerged. Fan tokens, for example, are crypto assets issued by clubs or organizations with large fan bases and combine collectible and utility features (7). In sport, fan tokens are used to facilitate fan participation and interaction, and may provide access to benefits such as voting opportunities, event privileges, or memorabilia (7). Many major football clubs, including FC Barcelona, Paris Saint-Germain, Manchester City, AC Milan, and Juventus, have launched fan tokens that are traded on cryptocurrency exchanges (8).

Despite these developments, research on cryptocurrencies in sport remains nascent and fragmented, and key themes have not been clearly mapped. Therefore, this study provides a thematic review of cryptocurrency applications in sport to identify major research themes and future directions. This study is essentially an exploratory literature-based investigation, and our research question is: What cryptocurrency-related research themes have been examined in the field of sport?

## 2 Materials and methods

### 2.1 Data sources

Web of Science (WoS) is the largest and most widely used academic databases, serving as the foundation for numerous literature-based analyses. Following the methodologies outlined in previous studies (9–12), we conducted our review using the core collection of WoS database. Meanwhile, we also used a snowball method to search the initially identified research in the Google scholar to check for their citations, references, and relevant publications.

### 2.2 Search strategies

Adapting the approach of other reviews related to cryptocurrency (13–16) and sport (15, 17–20), we executed a comprehensive search across titles, abstracts, and author keywords. The query combined the crypto/blockchain construct with the sport construct using Boolean operators and truncation. Specifically, we searched for (cryptocurrenc* OR token OR blockchain OR Bitcoin OR “digital currency”) AND (sport* OR “word cup” OR Olympic* OR championship). The time window extended from database inception to 2025/12/20, and the language was restricted to English.

### 2.3 Inclusion and exclusion criteria

We used the Population–Concept–Context (PCC) framework to delineate the scope of this review and to guide eligibility decisions (21).

Population (P) included sport fans, sports bettors, and relevant stakeholders such as sport organizations and clubs.

Concept (C) covered cryptocurrency-related technologies and digital assets, including both fungible tokens and non-fungible tokens (NFTs).

Context (C) referred to sport-industry–related activities and settings, such as sport media/marketing and event communication, betting and/or trading platforms, and applications or governance practices within specific countries or leagues.

It should be noted that this study is a theme-focused narrative (thematic) review aiming to map the evidence landscape; therefore, we did not restrict inclusion by study design or publication type. In addition to empirical studies, we considered case studies, conceptual/theoretical papers, opinion pieces, and editorials. Moreover, individual-level data were not required for eligibility: studies centered on sporting events, market transactions, platform mechanisms, or industry practices were also considered as long as they aligned with the review theme.

### 2.4 Analysis

We conducted a narrative review to identify the themes of the included studies. Although this approach is non-quantitative, it is well suited to synthesizing up-to-date knowledge on specific themes and providing a more nuanced understanding of the field (22). Meanwhile, Narrative reviews are qualitative research syntheses that describe the results of other studies without a dominant focus on the statistical significance of the findings (23). Theme development was primarily conducted using title-based coding to map the thematic landscape of the included literature. First, we conducted title-level open coding, assigning each paper 2–4 descriptive codes that captured the study’s primary focus as signaled in the title. Second, we performed pattern (axial) coding by grouping semantically overlapping codes into broader analytic categories. These categories were then reviewed and consolidated into five themes based on external distinctiveness (themes were largely different from each other).

## 3 Results

### 3.1 Literature selection

Our initial search in the Web of Science database yielded 127 potentially relevant studies. After removing duplicates and screening for relevance based on content, we included 20 articles. Thereafter, we employed the snowball method and included additional 10 articles. Eventually, a total of 30 articles were included for the review.

### 3.2 The characteristics of the included studies

The included literature (published between 2019 and 2025) was methodologically diverse, spanning quantitative financial analyses (event studies, intraday designs, and time-series models), cross-sectional surveys, broadcast content analyses, text-mining studies of user-generated data (e.g., app reviews and social media posts), qualitative interviews/case studies, and conceptual or ethics-focused papers (**Table 1**). Data sources ranged from token/cryptocurrency market and platform records to media-broadcast observations and questionnaire samples, with a noticeable concentration in European football contexts and several U.S.-based survey datasets, alongside a smaller number of studies from other regions and sports settings.

**Table 1:** Types of included studies and a summary of key findings.

Study	Design	Data source	Key points
Scharnowski et al. (7)	Event study/asset pricing	Fan token market data	Despite some non-monetary utility, fan tokens are highly speculative and resemble cryptocurrencies as financial assets.
Ante et al. (24)	Observational quantitative platform-data study	Fan token polls (N = 3,576)	Fan-token voting achieves relatively high engagement: ~50% of token holders participate on average. Participation varies with (dis-)agreement levels, poll type, sport sector, demographics, and club-level factors.
Manoli et al. (25)	Qualitative study	60 fan token consumers	Fan tokens function as “tokens of identity” for many fans—symbolic tools to express loyalty and strengthen engagement.
Chen (26)	Conceptual/critical essay	Spporters of Manchester City, Everton, and Crystal Palace	Fans’ discussions highlight contradictions of fan tokens as “engagement tools” versus instruments of financial speculation, and question cryptocurrency’s public utility.
Stegmann et al. (27)	Case study	A tokenized governance platform of a professional sport club	Governance tokens can foster engagement by involving fans in decision-making and enabling two-way communication with clubs.
Demir et al. (8)	Event-study	Fan tokens linked to football clubs	Both the losses and wins in the most prestigious European tournament, UEFA Champions League affect the fan token abnormal returns.
Lopez-Gonzalez and Griffiths (28)	Case study	FC Barcelona fan token	Fan tokens are framed as engagement tools (e.g., voting/experiences) but are also gamified digital items designed to keep users within providers’ apps.
Lopez-Gonzalez and Petrotta (29)	Cross-sectional survey	United States sports bettors (N≈525)	Observed significant overlap between experiencing gambling problems and consuming cryptocurrency trading, NFTs, and fan tokens.
Delfabbro et al. (30)	Narrative review	Not applicable	Crypto trading has structural features that may promote harm (e.g., high volatility, 24/7 availability, global market, strong social media/social influence, and non-fundamental events affecting prices).
Read and Smith (31)	Conceptual/ethics	Not applicable	Treats data as a form of capital that can be converted into economic capital via computational transformation, clarifying how digital sport business models create value.
Shao and Cheng (32)	Time-series (LASSO-VAR)	Chiliz (CHZ) and listed European football club stocks	Observed measurable interrelatedness/connectedness between CHZ and European football club stocks.
Torrance et al. (33)	Content analysis (broadcast frequency)	United Kingdom (EPL 2022/23 season; 10 matches)	Extremely high exposure: 20,941 gambling/gambling-like logos across 10 broadcasts; majority were gambling-only (64.1%), with substantial presence of cryptocurrency and financial trading logos (alone or combined categories).
Grubbs and Kraus (34)	Cross-sectional survey	United States (census-matched sample)	Regular crypto trading is more common among younger, more educated, higher-income men.
Delfabbro et al. (35)	Cross-sectional survey	Adults reporting at least monthly sports betting and/or crypto trading (N = 543)	Gambling involvement and stock trading are positively related to crypto trading. Problem gambling severity (PGSI) is positively associated with crypto trading intensity (time, trades, expenditure).
Mills and Nower (36)	Cross-sectional survey	Regular gamblers recruited via Amazon Mechanical Turk (N = 876)	Crypto trading is common: >50% of regular gamblers traded cryptocurrencies in the past year. Strong association with problem gambling severity.
Vidal-Tomás (37)	Multi-method financial market analysis	Fan tokens on Socios.com	Supporting a favorite team via fan tokens can lead to financial losses, whereas investing in Chiliz may allow traders to outperform the market.
Berkani et al. (15)	Systematic review	Not applicable	Catalogs a wide range of proposed blockchain use cases in sport (e.g., athlete data management, secure data sharing, event management, collectibles traceability), noting the field is often dominated by hype around fan tokens/NFTs.
Baldi et al. (38)	Text mining (sentiment + topic)	1,048 English-language user reviews	Identified five engagement drivers discussed in reviews: security, entertainment, customer service, user experience, and finances.
Ante et al. (39)	Intraday event study	8 fan tokens and 325 football matches	Fan token returns decline on average −0.8% during matches and a further −0.7% post-match. Identifies a pronounced “loss effect”: losses trigger larger negative reactions than the positive effects of wins.
Glebova and Mihail’Ova (40)	Conceptual/theoretical synthesis	Not applicable	Provided definitions and maps relationships among blockchain, fintech, cryptocurrencies, NFTs, smart contracts within professional sport.
Kang et al. (41)	Text mining (Twitter)	Tweets on Twitter (N = 23,445)	Sport NFT platforms appear to engage users via a mix of intrinsic and extrinsic motivations
Shuya et al. (42)	Cross-sectional survey	Brazil; football fans (n = 360)	TAM variables predict fan token adoption.
Vollero et al. (43)	Empirical quantitative study	Unknown	Meanings attached to fan-token activities → higher team brand identification and engagement.
Marques et al. (44)	Cross-sectional survey	Football fans	Perceived financial value is the strongest driver of purchase intention.
Shynkevich (45)	Observational event-study	Global bitcoin market	During penalty shootouts, Bitcoin trading volume and volatility drop. After shootouts end, trading rebounds.
Pelechrinis et al. (46)	Computational transaction-level analytics	NBA TopShot peer-to-peer transactions	Proposes a framework to label anomalous P2P NFT transactions using model-based anomaly probability.
Schlimm and Breuer (47)	Cross-sectional survey	Online sports consumers (n = 526)	Psychological involvement is the strongest predictor of interest in VE/Web3.
Agnese and Xiao (48)	Event study	Fan tokens of 4 major clubs	Fan tokens are primarily fan-sentiment–driven rather than a pure speculative crypto fad.
Park and Lee (49)	Qualitative case study	Fans of Kbollect	Identified five motives: financial rewards (most influential), exclusivity, hedonic motivation, sense of belonging, effort expectancy.
Schlimm et al. (50)	Cross-sectional survey	Supporters of sports teams/athletes	Social needs/wants show the strongest effect on NFT purchase intention.

### 3.3 Thematic review

**Figure 1:**
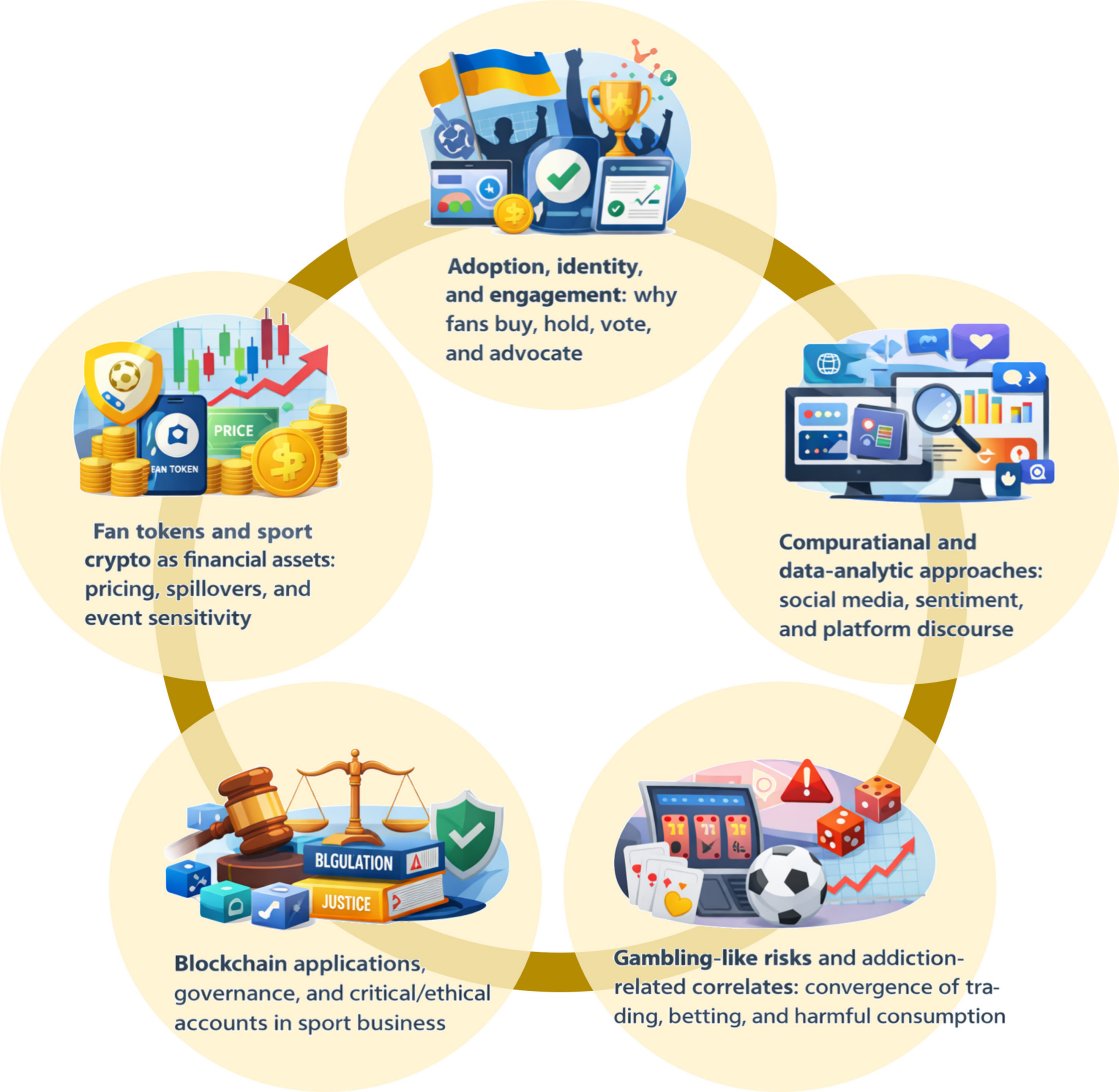
Research themes of the included studies.

Based on title–abstract coding of the 30 articles, the literature clusters into five recurring themes: (1) fan tokens as financial assets and sport-event-sensitive markets, (2) adoption, identity, and engagement mechanisms, (3) computational approaches to fan-token/NFT discourse and platform data, (4) blockchain applications, governance, and critical/ethical interpretations in sport business, and (5) gambling-like risks and addiction-related correlates surrounding crypto/NFT participation (**Figure 1**). The coding system can be found in **Supplementary Tables S1**–**S3**.

#### 3.3.1 Fan tokens and sport crypto as financial assets: pricing, spillovers, and event sensitivity

A prominent stream treats fan tokens as tradable digital assets whose returns co-move with broader crypto markets and respond to sport-related information shocks. Early market-facing work questions whether fan tokens meaningfully differ from other crypto assets, emphasizing their speculative profile despite purported “utility” narratives (8). Complementary pricing-oriented research argues that fan tokens often behave like high-volatility crypto instruments embedded in speculative cycles (7), while broader conceptual–economic discussion situates fan tokens within blockchain-enabled sport finance and stakeholder expectations (37). Event-linked studies further document how match outcomes and sporting performance map onto intraday fan token returns, strengthening the argument that sport information is priced—yet within a market structure still dominated by crypto-like dynamics (39). Beyond match events, sport–crypto connectedness research explores spillovers between sport-related crypto assets and traditional football equities (32). Finally, event–market analogies extend into “sport as a sentiment shock” contexts, showing how discrete sport events can align with crypto trading patterns (45).

#### 3.3.2 Adoption, identity, and engagement: why fans buy, hold, vote, and advocate

A second theme centers on consumer meaning-making and engagement mechanisms, examining how fan identity, perceived value, and perceived risk shape adoption and downstream behaviors. Extended technology-acceptance work models how identity and risk perceptions jointly influence acceptance and behavioral intentions in sport-fan contexts (42). In parallel, engagement-focused studies argue that the “meaning” fans attach to fan-token activities supports identification with team brands and co-creation pathways, offering a marketing logic for token-based engagement strategies (43). Purchase-intention models in sport fandom emphasize perceived value and trust/risk appraisals as predictors of buying behavior and advocacy outcomes (44). Behavioral participation is also approached directly through platform governance features: fan-token voting participation is analyzed as a measurable form of engagement whose patterns vary across polls and contexts (24). Related work questions whether tokenization in leagues truly delivers engagement “for real,” contrasting promised relational value with observed participation and market behavior (48).

This stream also expands from fan tokens to sports NFTs as adjacent engagement/consumption assets. Studies of virtual environments and Web3 activations examine consumers’ interest in immersive or digitally mediated sport experiences that may normalize token/NFT touchpoints (47). Sports NFT research further decomposes purchase intention into consumer values and needs, positioning NFT buying as a multi-motive decision rather than purely speculative (50). Case-based evidence additionally compares NFT holders and non-holders to map motivational differences and platform-specific consumption styles (49). Finally, leisure-theory framing highlights the dual nature of fan tokens as identity projects and investment practices, suggesting that “serious leisure” logics can coexist with financial motives (25).

#### 3.3.3 Computational and data-analytic approaches: social media, sentiment, and platform discourse

A third theme leverages machine learning, deep learning, and computational text analytics to understand perceived values/risks and engagement narratives at scale. One line applies machine and deep learning to sport NFT tweet data to classify or infer perceived value and risk signals embedded in public discourse (41). Another analyzes fan-token platform content through sentiment and deep-learning-driven content analysis to characterize community narratives and interaction patterns (38). Together, these studies show how fan-token and NFT ecosystems generate large volumes of naturally occurring text data that can be mined for perceptions, concerns, and drivers of participation—offering a methodological bridge between marketing/consumer research and computational social science.

#### 3.3.4 Blockchain applications, governance, and critical/ethical accounts in sport business

A fourth theme addresses what blockchain is used for in sport (beyond trading), how governance is structured, and how ethical/political economy concerns are raised. Systematic review work catalogs sport blockchain use cases across stakeholders and highlights a recurring implementation gap—many proposed applications exist conceptually but are less often validated through robust prototypes or evaluations (15). Stakeholder-oriented conceptual analysis similarly maps how blockchain, fintech, NFTs, and related tools may reconfigure roles, value creation, and strategic priorities in professional sport (40).

Crucially, critical scholarship interrogates the political economy of these assets. Fan tokens and sport NFTs are framed as part of crypto-capitalist logics that may intensify commodification of fandom, recasting “engagement” as monetizable participation under platform capitalism (26). From a business-model ethics perspective, digital sport models that treat data as capital raise questions about governance, surveillance, and responsible commercialization, especially when fan engagement is entwined with extractive data practices (31). Marketing-oriented conceptual work further problematizes “tokenization” as potentially hype-driven while still identifying conditions under which it could function as a genuine engagement platform (27).

#### 3.3.5 Gambling-like risks and addiction-related correlates: convergence of trading, betting, and harmful consumption

A fifth theme evaluates risk pathways and harm correlates at the intersection of crypto trading, sport betting, and digital-asset consumption. Foundational evidence indicates that cryptocurrency trading is associated with gambling engagement and problem gambling indicators, suggesting partial behavioral overlap between speculative trading and gambling-related harm profiles (35). Psychological work further identifies risk and protective factors in cryptocurrency trading, supporting the view that individual differences and behavioral tendencies shape vulnerability (30). Among regular gamblers, early findings warn that cryptocurrency trading can represent an additional risk factor for gambling problems (36). Population-matched evidence also links cryptocurrency involvement with broader addictive behaviors, underscoring that harms may generalize beyond gambling-only frameworks (34).

In sport-specific contexts, research identifies “gambling-like” design features in fan tokens, emphasizing structural similarities that may encourage risky engagement patterns (28). Complementary correlational work among US sports bettors connects consumption of cryptocurrencies, NFTs, and fan tokens with gambling severity, suggesting convergent risk environments for bettors who engage with these assets (29). Marketing ecology studies extend the harm lens to sport media environments by documenting the prominence of gambling, crypto, and trading app promotion within elite football broadcasts, raising concerns about normalization and exposure effects (33). Finally, market integrity work on NFT marketplaces develops methods to detect anomalous trades—relevant not only for fraud and manipulation but also for understanding the ecosystem conditions that can amplify consumer risk (46).

## 4 Discussion

This study conducted a thematic review based on the Web of Science (WoS) database, surveying key research themes on cryptocurrency in the sports domain. It identified major themes such as gambling-like risks and addiction-related correlates, suggesting that these areas represent the primary directions of current research. This review is intended to map and summarize research themes rather than to evaluate the strength, importance, or statistical significance of findings within each theme. Given the limited and methodologically diverse evidence base, we did not perform a formal quality appraisal or quantitative synthesis. Accordingly, the results reported in the original studies and cited in our Results section should be interpreted with caution: they should not be taken as definitive or high-certainty evidence, but rather as indicative signals that help generate hypotheses, highlight knowledge gaps, and provide directions for future research and responsible practice.

### 4.1 General discussion

The adoption of cryptocurrencies remains exploratory. With technological maturation and the emergence of new applications, future research in this area is expected to diversify and offer novel theoretical and practical perspectives.

Based on our five themes, the existing literature collectively portrays an overarching picture of the “assetization of sports fandom.” At the industry and institutional level, blockchain and tokenization are framed as scalable digital infrastructures that link clubs, platforms, and fans, and that enable new business models through the re-organization of data and rights. At the interaction level, voting, co-creation, and Web3 activations concretize “engagement” into behaviors that can be designed, quantified, and operationalized. At the individual level, adoption and purchase studies further show that fans’ entry into this ecosystem is rarely driven by a single motive: identity affirmation, community belonging, experiential value, and investment expectations often coexist, and are moderated by trust and risk appraisals. Meanwhile, once these mechanisms are embedded in real markets, fan tokens and sports NFTs quickly display characteristics typical of financial assets—prices are highly sensitive to sporting events and attention dynamics, and volatility and connectedness mean that these products both serve “fan relationships” and become incorporated into broader crypto-trading logics. Finally, the gamblingization and consumer-protection theme reminds us that this coupling of “engagement–trading” is not value-neutral: when reward structures, marketing exposure, and highly volatile trading environments overlap, engagement may be pushed toward more intense speculative consumption, intersecting with addiction-related or problem-gambling risks for some users. Taken together, fan tokens/NFTs are not merely new technologies or products; rather, they resemble a socio-technical-financial assemblage that simultaneously bundles identity, platform governance, market mechanisms, and risk exposure. Accordingly, understanding them requires attention to the engagement logic of sport management and marketing, the pricing logic of financial markets, and the risk logic of public health and ethical governance.

At the same time, these five themes also reveal several gaps that merit future research. First, studies within each theme often treat their own outcome variables as endpoints (e.g., intention, participation rates, returns, risk correlates), yet rarely address, within a single framework, the causal and transformational relationships among “engagement motives–trading behaviors–risk consequences.” For example, it remains unclear whether voting/right-design or incentive structures shift some users from relationship-based engagement toward high-frequency speculation, and under what conditions such a transition occurs. This gap calls for more integrative frameworks and longitudinal designs to trace pathways leading to adverse outcomes. In addition, although the gamblingization and consumer-protection literature has identified “gambling-like” product features of fan tokens (28) and has observed associations between crypto trading and problem gambling/addiction-related indicators in both gambler and general-population samples (34, 36), the evidence base remains largely correlational, and actionable discussions of regulatory frameworks, platform governance, and design responsibilities are relatively limited. Future studies could systematically compare platforms or rule versions and evaluate the effects of consumer-protection measures—such as the strength of risk warnings, underage restrictions, cooling-off periods or frequency limits, “de-gamblingization” adjustments to rewards and tasks, and disclosure standards—on high-frequency trading, impulsive trading, and problem-gambling indicators, thereby producing an operational checklist for regulation and platform design.

Finally, we would like to call for greater attention to some “hidden corners” of this research area. As our results indicate, the existing literature is concentrated primarily in Europe and North America. In fact, there remains a substantial population of cryptocurrency holders in other countries, such as China. In China, electronic and mobile payments are highly prevalent (51), yet cryptocurrency trading is not supported by law (52, 53). This contrast may make public understanding and perceptions of cryptocurrencies particularly complex. As a result, Chinese citizens’ motivations for using cryptocurrencies and their related behaviors may differ in important ways from those observed in other regions (54, 55). Similarly, many Global South countries (low- and lower-middle-income countries/developing economies) also warrant attention. Because financial infrastructures, regulation, and consumer protections often differ in Global South settings, engagement may reflect more instrumental needs (e.g., cross-border payments or alternative financial channels) alongside greater exposure to information asymmetry, fraud, and volatility-related losses. Future research should therefore prioritize descriptive and comparative studies in Global South contexts, complemented by qualitative work to capture localized trust, legitimacy, and risk-perception mechanisms.

### 4.2 Regulatory recommendations

To improve consumer protection and preserve the integrity of sport ecosystems, policy and industry stakeholders could operationalize the following measures for sport-linked crypto products (including fan tokens):

Clear product classification and scope: Clarify whether fan tokens function primarily as consumer utility products, financial instruments, or mixed products, and apply corresponding conduct and disclosure obligations (including market-abuse controls where trading occurs).
Stronger marketing safeguards in sport settings: Require prominent, standardized risk warnings (volatility, loss probability, lack of guarantees), prohibit misleading “engagement” framing that obscures speculative features, and ensure promotions are fair, clear, and not misleading—particularly for campaigns embedded in sport broadcasts and club channels.
Platform governance and market integrity: Enforce surveillance and controls for insider trading and market manipulation risks around sport events, and require robust listing standards, conflict-of-interest disclosures, and transparent tokenomics for club-linked tokens.
Built-in consumer protection tools: Encourage “safety-by-design” features (spend/loss limits, cooling-off prompts, reality checks) and evaluate them using platform experiments or quasi-experimental designs to identify scalable harm-reduction approaches.

### 4.3 Limitations

This study is based on an analysis of existing academic literature. Because the application of cryptocurrencies in sport remains in its early developmental stage, the number of relevant studies is still limited. Consequently, the present findings may not fully capture the latest developments, including recent innovations implemented by sport organizations or emerging commercial practices. Future investigations could incorporate additional data sources—such as industry white papers, corporate announcements, or social media data—to provide a more comprehensive and up-to-date perspective.

This review included only publications indexed in major databases (Web of Science core databases). Relevant works indexed in other repositories may therefore have been omitted. Additionally, the analysis was restricted to English-language publications for practical reasons, leaving research in other languages unexplored. Future bibliometric and thematic reviews should consider expanding linguistic and database coverage to achieve a more global and inclusive understanding of cryptocurrency adoption in the sport industry.

The current evidence base is heavily concentrated in European football and U.S. samples, with limited coverage of other sports and non-Western contexts; therefore, the global generalizability of our findings is constrained and context-specific mechanisms may be underrepresented.

A key limitation is that the peer-reviewed evidence base on cryptocurrencies in sport remains small, and the included studies are highly heterogeneous in design, data sources, and analytic approaches (e.g., market event studies/time-series analyses, platform analytics, broadcast content analyses, cross-sectional surveys, qualitative and conceptual work). This heterogeneity precludes meaningful quantitative aggregation and limits cross-study comparability; therefore, our thematic findings should be interpreted as an overview of emerging themes rather than as firm conclusions or in-depth inferences about specific effects or outcomes.

This work is primarily literature-driven; therefore, several viewpoints and proposed directions reflect the current evidence base and our synthesis, rather than conclusions validated through expert consensus. Given the early-stage and rapidly evolving nature of this theme, future research should incorporate structured expert methods (e.g., Delphi technique and focus groups) to critically assess, refine, and prioritize the most valuable themes and actionable research agendas in sport-related cryptocurrencies.

## 5 Conclusions

This study draws on the WoS Core Collection and snowball searching to include 30 English-language studies (2019–2025) on “cryptocurrency/blockchain in sport,” and thematically maps the evidence using title–abstract coding. Overall, the literature depicts an ongoing “assetization” of sport fandom: Web3 products such as fan tokens and sport NFTs are marketed as quantifiable, designable, and operable engagement tools (e.g., voting, perks, immersive experiences), yet in real markets they quickly exhibit financial-asset characteristics—high volatility, event sensitivity, and linkages with the broader crypto market—and may intersect with speculative consumption, gambling-like mechanisms, and addiction-related risks. Within this overarching picture, the research can be classified into five stable thematic strands: pricing and event reactions as financial assets; adoption–identity–engagement mechanisms; computational and platform-data approaches to social discourse; blockchain applications with governance and ethical critique; and gambling-like risks and addiction-related correlates.

## Data Availability

The original contributions presented in the study are included in the article/**Supplementary Material**. Further inquiries can be directed to the corresponding author.
