# Supplementary material for: Cryptocurrency in sport: a thematic review
Source: Front Psychiatry. 2026 Jan 28;16:1745490. doi: 10.3389/fpsyt.2025.1745490 (PMC12891126; doi:10.3389/fpsyt.2025.1745490)
Supplement: Supplementary file 1 [file Table1.docx]

Supplementary Material

Table S2. Codebook (final open codes, definitions, and title triggers)

| **Code family** | **Open code** | **Definition (title-level)** | **Typical title triggers** |
| --- | --- | --- | --- |
| Finance / market | **C-PRICING** | Pricing/returns/volatility/speculation/trading as focal outcomes | returns, volatility, speculation, trading, market |
| Finance / market | **C-SPILLOVER** | Spillovers/connectedness/network linkages across markets/assets | connectedness, spillover, network, stocks, VAR |
| Finance / market | **C-EVENT** | Sport event/performance sensitivity used as a driver/shock | football games, sporting performance, penalty shootouts |
| Finance / market | **C-INTEGRITY** | Market integrity issues, abnormal/anomalous trades | anomalous trades, spotting, manipulation |
| Adoption / engagement | **C-ADOPTION** | Adoption/acceptance/behavioral intention (often TAM-related) | acceptance, TAM, behavioural intention |
| Adoption / engagement | **C-MOTIVES** | Motives/values/needs/purchase intention | motives, values, needs, purchase intention |
| Adoption / engagement | **C-IDENTITY** | Identity/identification/meaning-making/leisure framing | identity, identification, meaning, serious leisure |
| Adoption / engagement | **C-PARTICIPATION** | Engagement/holding/voting/advocacy/co-creation/Web3 interest | engagement, participation, voting, co-creation, Web3, activations |
| Computational discourse | **C-COMP** | Computational/data-analytic approaches applied to social media/platform discourse | machine learning, deep learning, sentiment, content analysis, tweets, platform discourse |
| Sport business / governance / ethics | **C-USECASES** | Systematic review / use cases / mapping applications | systematic review, use cases |
| Sport business / governance / ethics | **C-STAKE** | Stakeholder approach / ecosystem framing / fintech culture | stakeholder, ecosystem, fintech culture |
| Sport business / governance / ethics | **C-ETHICS** | Ethics/datafication (e.g., data as capital, ethical implications) | ethical, data as capital, implications |
| Sport business / governance / ethics | **C-CRIT** | Critical political economy (e.g., crypto-capitalism, commodification) | crypto-capitalism, hypercommodification, contesting |
| Gambling / harm | **C-HARM** | Gambling-like features, addiction, problem gambling, gambling severity | gambling-like, addictive, problem gambling, severity |
| Gambling / harm | **C-EXPOSURE** | Marketing/advertising exposure in sport media contexts | marketing, frequency analysis, logos |

Table S2. Deterministic theme-assignment rules

| **Theme #** | **Theme label (as used in the review)** | **Primary decision rule (dominant title focus)** | **Primary code triggers** |
| --- | --- | --- | --- |
| 1 | Fan tokens and sport crypto as financial assets: pricing, spillovers, and event sensitivity | Assign if the title foregrounds **returns/pricing**, **connectedness/spillovers**, **event sensitivity**, or **market integrity** | C-PRICING, C-SPILLOVER, C-EVENT, C-INTEGRITY |
| 2 | Adoption, identity, and engagement: why fans buy, hold, vote, and advocate | Assign if the title foregrounds **adoption/intent**, **motives/values/needs**, **identity/meaning**, or **engagement/voting/advocacy** (and computational analytics is *not* the central contribution) | C-ADOPTION, C-MOTIVES, C-IDENTITY, C-PARTICIPATION |
| 3 | Computational and data-analytic approaches: social media, sentiment, and platform discourse | Assign if the title foregrounds **computational analytics of discourse** (ML/DL/sentiment/content analysis/tweets/platform discourse) | C-COMP |
| 4 | Blockchain applications, governance, and critical/ethical accounts in sport business | Assign if the title foregrounds **use-case mapping/systematic review**, **stakeholder/ecosystem framing**, or **ethical/critical** interpretations | C-USECASES, C-STAKE, C-ETHICS, C-CRIT |
| 5 | Gambling-like risks and addiction-related correlates: convergence of trading, betting, and harmful consumption | Assign if the title foregrounds **gambling-like/addiction/problem gambling** or **marketing exposure** in sport contexts | C-HARM, C-EXPOSURE |

Table S3. Full audit trail

| **#** | **Paper (short title)** | **Extracted open codes (from title cues)** | **Assigned theme** |
| --- | --- | --- | --- |
| 1 | Application of ML/DL on Sport NFT Tweets (values/risks) | C-COMP; C-MOTIVES | Theme 3 |
| 2 | Are Fan Tokens Fan Tokens? | C-PRICING | Theme 1 |
| 3 | Blockchain Use Cases in the Sports Industry: A Systematic Review | C-USECASES | Theme 4 |
| 4 | Blockchain, Sport and Fan Tokens | C-STAKE; C-USECASES (broad mapping cue) | Theme 4 |
| 5 | Contesting Fan Tokens Under Crypto-Capitalism… | C-CRIT | Theme 4 |
| 6 | Cryptocurrency and Addictive Behaviors in a Census-Matched US Sample | C-HARM | Theme 5 |
| 7 | Cryptocurrency Trading, Gambling and Problem Gambling | C-HARM | Theme 5 |
| 8 | Data as Capital and Ethical Implications in Digital Sport Business Models | C-ETHICS | Theme 4 |
| 9 | Identity and Perceived Risk on Technology Acceptance… (Extended TAM) | C-ADOPTION; C-IDENTITY (identity cue); (risk cue aligned with adoption) | Theme 2 |
| 10 | Football Fan Tokens’ Influence on Engagement (meaning/brand ID/cocreation) | C-PARTICIPATION; C-IDENTITY | Theme 2 |
| 11 | Factors Influencing Fan Token Purchase Intent… | C-MOTIVES; C-ADOPTION | Theme 2 |
| 12 | Fan Tokens: Sports and Speculation on the Blockchain | C-PRICING | Theme 1 |
| 13 | Football Fan Tokens as “Serious Leisure”… (identity & investment) | C-IDENTITY; C-MOTIVES | Theme 2 |
| 14 | EPL Marketing: Gambling/Crypto/Trading App Logos (frequency analysis) | C-EXPOSURE; C-HARM | Theme 5 |
| 15 | Gambling-Like Digital Assets and Gambling Severity (sports bettors) | C-HARM | Theme 5 |
| 16 | Gambling-Like Features in Fan Tokens | C-HARM | Theme 5 |
| 17 | Hype or Opportunity? Tokenization as Engagement Platform in Sport Marketing | C-PARTICIPATION; C-STAKE | Theme 2 |
| 18 | New Currencies and New Values in Professional Sports (stakeholder approach) | C-STAKE | Theme 4 |
| 19 | Penalty Shootouts and Bitcoin Trading | C-EVENT; C-PRICING | Theme 1 |
| 20 | Preliminary Findings on Cryptocurrency Trading Among Regular Gamblers… | C-HARM | Theme 5 |
| 21 | Sentiment and DL Content Analysis of a Digital Fan Token Platform | C-COMP; C-PARTICIPATION (platform discourse cue) | Theme 3 |
| 22 | Spotting Anomalous Trades in NFT Markets: NBA Topshot | C-INTEGRITY; C-PRICING | Theme 1 |
| 23 | Taking Fan Engagement to a New Level: Web3 Activations (consumer interest) | C-PARTICIPATION | Theme 2 |
| 24 | Football Games/Performance and Intra-Day Fan Token Returns | C-EVENT; C-PRICING | Theme 1 |
| 25 | Psychology of Cryptocurrency Trading: Risk and Protective Factors | C-HARM | Theme 5 |
| 26 | Time-Varying Connectedness: Sport Crypto and Football Stocks (LASSO-VAR) | C-SPILLOVER | Theme 1 |
| 27 | Tokenization in Soccer Leagues: Is Fan Engagement for Real? | C-PARTICIPATION | Theme 2 |
| 28 | Motives/Consumption: Sports NFT Holders vs Non-Holders (Kbollect) | C-MOTIVES; C-ADOPTION | Theme 2 |
| 29 | Voting Participation and Engagement in Blockchain-Based Fan Tokens | C-PARTICIPATION (voting participation cue) | Theme 2 |
| 30 | Why Do Consumers Buy Sports NFTs? (values/needs → purchase intention) | C-MOTIVES | Theme 2 |
